# Evaluation of Primers Targeting the Diazotroph Functional Gene and Development of NifMAP – A Bioinformatics Pipeline for Analyzing *nifH* Amplicon Data

**DOI:** 10.3389/fmicb.2018.00703

**Published:** 2018-04-30

**Authors:** Roey Angel, Maximilian Nepel, Christopher Panhölzl, Hannes Schmidt, Craig W. Herbold, Stephanie A. Eichorst, Dagmar Woebken

**Affiliations:** Division of Microbial Ecology, Department of Microbiology and Ecosystem Science, Research Network ‘Chemistry meets Microbiology’, University of Vienna, Vienna, Austria

**Keywords:** nitrogen fixation, primer evaluation, *nifH* gene, Illumina amplicon sequencing, NifMAP

## Abstract

Diazotrophic microorganisms introduce biologically available nitrogen (N) to the global N cycle through the activity of the nitrogenase enzyme. The genetically conserved dinitrogenase reductase (*nifH*) gene is phylogenetically distributed across four clusters (I–IV) and is widely used as a marker gene for N_2_ fixation, permitting investigators to study the genetic diversity of diazotrophs in nature and target potential participants in N_2_ fixation. To date there have been limited, standardized pipelines for analyzing the *nifH* functional gene, which is in stark contrast to the 16S rRNA gene. Here we present a bioinformatics pipeline for processing *nifH* amplicon datasets – NifMAP (“*NifH* MiSeq Illumina Amplicon Analysis Pipeline”), which as a novel aspect uses Hidden-Markov Models to filter out homologous genes to *nifH*. By using this pipeline, we evaluated the broadly inclusive primer pairs (Ueda19F–R6, IGK3–DVV, and F2–R6) that target the *nifH* gene. To evaluate any systematic biases, the *nifH* gene was amplified with the aforementioned primer pairs in a diverse collection of environmental samples (soils, rhizosphere and roots samples, biological soil crusts and estuarine samples), in addition to a *nifH* mock community consisting of six phylogenetically diverse members. We noted that all primer pairs co-amplified *nifH* homologs to varying degrees; up to 90% of the amplicons were *nifH* homologs with IGK3–DVV in some samples (rhizosphere and roots from tall oat-grass). In regards to specificity, we observed some degree of bias across the primer pairs. For example, primer pair F2–R6 discriminated against cyanobacteria (amongst others), yet captured many sequences from subclusters IIIE and IIIL-N. These aforementioned subclusters were largely missing by the primer pair IGK3–DVV, which also tended to discriminate against Alphaproteobacteria, but amplified sequences within clusters IIIC (affiliated with Clostridia) and clusters IVB and IVC. Primer pair Ueda19F–R6 exhibited the least bias and successfully captured diazotrophs in cluster I and subclusters IIIE, IIIL, IIIM, and IIIN, but tended to discriminate against Firmicutes and subcluster IIIC. Taken together, our newly established bioinformatics pipeline, NifMAP, along with our systematic evaluations of *nifH* primer pairs permit more robust, high-throughput investigations of diazotrophs in diverse environments.

## Introduction

Nitrogen gas (N_2_) fixing microorganisms (diazotrophs) are one of the most ecologically important functional guilds on Earth, providing the primary natural source for nitrogen to ecosystems through biological N_2_ fixation (BNF; Fowler et al., 2013). Isotopic evidence suggests that BNF has emerged as early as ca. 3.2 Gyr ago (Stüeken et al., 2015). It is thought to have evolved in an anaerobic archaeon and was later transferred to an aerobic bacterium (Boyd et al., 2015). Considering the time scale of the BNF evolution and the importance of N_2_ fixation, it is not surprising that the genetic potential to perform N_2_ fixation (i.e., the *nif* genes) is found widely among different branches in the phylogenetic trees of archaea and bacteria. It is estimated that 6–15% of all sequenced microbial genomes harbor the minimum number of *nif* genes to provide them with the genetic capacity to fix N_2_ (Dos Santos et al., 2012; Boyd et al., 2015), making diazotrophs one of the most diverse functional guilds.

Among all known diazotrophs, N_2_ fixation is mediated by the nitrogenase enzyme complex along with ca. 20 functional and regulatory genes that are organized in several operons termed together the *Nif* regulon (Rubio and Ludden, 2002). Of those genes, the *nifH* gene encoding for the dinitrogenase reductase is considered one of the most genetically conserved genes in the regulon and has been traditionally used as a marker gene for studying the genetic diversity of diazotrophs in nature (Zehr et al., 2003; Gaby and Buckley, 2011). In addition to the canonical MoFe nitrogenase that is shared among all known diazotrophs, some diazotrophs possess in their genome one of the two alternative versions of nitrogenase, which use either VFe (*Vnf*) or FeFe (*Anf*) as metal cofactors (Raymond et al., 2004).

Based on phylogenetic analysis, the *nifH* genes typically form four clusters, termed clusters I–IV (Zehr et al., 2003; Raymond et al., 2004). Most *nifH* sequences fall into cluster I, which is composed almost entirely of sequences from the canonical MoFe nitrogenase. This cluster typically consists of sequences from Cyanobacteria, *Frankia*, Proteobacteria, and some are affiliated with Clostridia, Bacilli, and Nitrospirae. In addition, cluster I contains some sequences of the alternative nitrogenase *vnfH*. Cluster II comprises nearly all sequences of the alternative *vnfH* nitrogenase as well as all the known sequences of the second alternative nitrogenase, *anfH*. In addition, the *nifH* genes of some Archaea also fall into cluster II. Cluster III consists mostly of *nifH* sequences of anaerobic bacteria and archaea such as methanogens, spirochetes, sulfate reducers, non-sulfur purple bacteria, green sulfur bacteria, acetogens and Clostridia. Cluster IV was considered until recently to only contain “uncharacterized” or “non-functional” *nifH* sequences, but in 2015 the first isolate of a diazotroph containing an active nitrogenase belonging to cluster IV was cultivated from a termite gut (Zheng et al., 2016). While it has been shown in several pure cultures that most of the known cluster IV nitrogenases do not encode for a protein involved in N_2_ fixation (e.g., Staples et al., 2007), it is certainly possible that some of the environmental sequences in this cluster do encode for active nitrogenases.

The dinitrogenase reductase gene is found in nearly every environment studied so far (Gaby and Buckley, 2011), and environmental genetic surveys of the *nifH* gene have produced extensive datasets encompassing several tens of thousands of unique sequences (Gaby and Buckley, 2014; Heller et al., 2014), and novel sequences continue to be discovered. Such PCR-based surveys continue to serve as an important tool for studying the diazotroph diversity and, if the *nifH* transcripts are targeted (via cDNA), can also reveal transcription patterns in the environment. Yet, the choice of primer pair can have a significant impact on the extent of diversity that is uncovered. Since the first degenerate primers were used to amplify environmental DNA (Kirshtein et al., 1991), several dozens of primer pairs targeting the *nifH* gene have been designed to serve as group-specific or general *nifH* primers. In 2012, Gaby and Buckley published an extensive review of all known primers for *nifH*, and evaluated their performance *in silico* using a comprehensive database of *nifH* sequences (Gaby and Buckley, 2012). Through their analysis, several primer pairs were identified for their potential to capture the largest diversity of *nifH* sequences and were tested for their ability to produce a PCR product from DNA from several diazotrophic strains and two soil samples (Gaby and Buckley, 2012). However, the extent to which these primers are able to capture *nifH* diversity in environmental samples and their potential preferences of amplification have not been tested. Furthermore, the majority of the *nifH* sequences in public databases have sequence data between positions 100 and 500 bases (positions are relative to *Azotobacter vinelandii*), making it challenging to perform *in silico* coverage estimates of primer pairs flanking this region.

Given the importance of exploring the diazotrophic communities in the environment and the need for bioinformatics pipelines for analysis of the *nifH* gene, we developed a bioinformatics pipeline for processing *nifH* amplicon datasets derived from the MiSeq Illumina sequencing platform – NifMAP (“*NifH* MiSeq Illumina Amplicon Analysis Pipeline”). Using this pipeline, our further goal of this study was to evaluate the performance of general primer sets – selected based on their high *in silico* coverage for amplifying *nifH* – via high-throughput sequencing of a mock community and across a diverse collection of environmental samples. We discuss the performance of the tested primer pairs and provide a standard operating procedure for analyzing *nifH* genes using high-throughput sequencing.

## Materials and Methods

### Primers Used in This Study

Four forward primers: Nh21F, Ueda19F, F2, and IGK3 and four reverse primers: *nifH*1, *nifH*3, R6, and DVV were chosen following the *in silico* analysis of Gaby and Buckley (2012) (**Table 1**). The forward primers Nh21F, Ueda19F, and F2 and the reverse primers *nifH*1, *nifH*3, and R6 were tested in all nine combinations. In addition, the primer pair IGK3–DVV was tested, as it was suggested as the best performing primer pair in Gaby and Buckley (2012).

**Table 1:** Summary of the *nifH* primers used in this study.

Primer name	Direction^1^	Sequence (5′ to 3′)	Position^2^	Reference	Coverage (%) with mismatches^3^		
					0	1	2
Nh21F	F	GCI WTY TAY GGN AAR GG	19–35	Deslippe et al., 2005	81%	94%	98%
Ueda19F	F	GCI WTY TAY GGI AAR GGI GG	19–38	Ueda et al., 1995	81%	94%	98%
F2	F	TGY GAY CCI AAI GCI GA	115–131	Marusina et al., 2001	87%	91%	98%
IGK3	F	GCI WTH TAY GGI AAR GGI GGI ATH GGI AA	19–47	Ando et al., 2005	83%	96%	98%
*nifH*1	R	ADN GCC ATC ATY TCN CC	406–476	Zehr and McReynolds, 1989	85%	93%	95%
*nifH*3	R	ATR TTR TTN GCN GCR TA	478–494	Zani et al., 2000	89%	93%	96%
R6	R	GCC ATC ATY TCI CCI GA	457–473	Marusina et al., 2001	90%	93%	96%
DVV	R	ATI GCR AAI CCI CCR CAI ACI ACR TC	388–413	Ando et al., 2005	96%	98%	99%

*^*1*^Either forward (F) or reverse (R).*

*^*2*^Position is relative to *A. vinelandii *nifH** (GenBank ACCN# M20568).*

*^*3*^Matching of the primers to *nifH* sequences in the “*nifH*_2014April04.arb” database (Heller et al., 2014; 41,231 sequences in the database). The number of sequences in the database that extended into each respective primer matching region are: Nh21F (2156); Ueda19F (2156); F2 (14,529); IGK3 (2156); *nifH*1 (9788); *nifH*3 (5523); R6 (13,673); and DVV (30,142). The percentages refer to the fraction of these aforementioned sequences covered by the primers with either 0, 1, or 2 mismatches.*

### Samples Used in This Study

Thirteen different samples were used in this study: soil samples from (a) an Austrian beech forest (Rasche et al., 2011) and (b) an Austrian meadow (Angeringer and Karrer, 2008); (c) rhizosphere and (d) root of *Arrhenatherum elatius* (tall oat-grass) from an Austrian grassland site (Pötsch et al., 2013); (e) rhizosphere and (f) root of *Oryza sativa* (wetland rice, grown in the greenhouse on paddy soil from Vercelli, Italy); biological soil crusts (BSCs) from (g) a coastal, sub-arctic crust, Sweden, (h) a temperate crust, Germany, (i) a high Alpine crust, Austria, (j) a semiarid crust, Spain (Büdel et al., 2014) and (k) an arid crust, Israel (Angel and Conrad, 2009); and two estuarine samples from (l) the Great Belt that is separating the North Sea and the Baltic Sea and (m) the Roskilde Fjord, Denmark (Bentzon-Tilia et al., 2015).

### DNA Extraction

With the exception of the water samples, DNA was extracted from 0.4 g of soil (or 0.2–0.4 g root and rhizosphere samples) using modification of a standard bead-beating protocol in the presence of a CTAB buffer and phenol, according to a previously published phenol/chloroform-based extraction protocol (Angel, 2012). Following extraction, samples were purified using OneStep^TM^ PCR Inhibitor Removal Kit (Zymo, Irvine, CA, United States). DNA from the water samples were also obtained with a phenol/chloroform-based protocol, as described in Boström et al. (2004). DNA was extracted from biological replicates: the Austrian beech forest (*n* = 6); Austrian meadow soil (*n* = 6); BSCs (*n* = 2/type); root and rhizosphere samples (*n* = 3/type); and estuarine samples (*n* = 2).

### PCR Amplification and Sequencing

For an initial pre-screening of the different *nifH*-primer combinations, DNA from the beech forest and meadow soils were used for PCR amplification of the *nifH* gene fragment, and the PCR products were evaluated using agarose gel electrophoresis. Amplifications were performed in 25 μl volume using the following mixture: 2.5 μl of 10× DreamTaq Green Buffer, 2 mM MgCl_2_, 0.2 mM of each nucleotide dNTP mixture, 0.08 μg μl^-1^ of BSA, 0.625 U of DreamTaq Green DNA Polymerase (all from Thermo Fisher Scientific, Waltham, MA, United States) and 0.8 μM of each primer (Thermo Fischer Scientific, Waltham, MA, United States) and 1 μl of DNA template. The primers were designed to include a universal 16 bp head sequence at their 5′ end for subsequent barcoding (Herbold et al., 2015). The following program was used for amplification: 94°C for 5 min followed by 35 cycles of 94°C for 30 s, 52°C for 45 s, and 72°C for 30 s, and a single step of final elongation at 72°C for 10 min. Sequencing of amplified *nifH* genes was done using multiplexed barcoded amplicon sequencing on an Illumina MiSeq platform (Illumina, San Diego, CA, United States), as described previously (Herbold et al., 2015). First-step PCR amplifications were performed in triplicates of 25 μl each, using the mixture and conditions described above, except that PCRs were done in 25 cycles. Following PCR amplification, samples were purified using ZR-96 DNA Clean-up Kit^TM^ (Zymo, Irvine, CA, United States) and 3 μl from the purified sample was used for a second PCR reaction, which was 50 μl in volume and contained the following mixture: 5 μl of 10× DreamTaq Green Buffer, 2 mM MgCl_2_, 0.2 mM of each nucleotide dNTP mixture, 0.08 μg μl^-1^ of BSA, 1.25 U of DreamTaq Green DNA Polymerase (all from Thermo Fisher Scientific, Waltham, MA, United States) and 0.4 μM of a barcode primer, which also contained a universal 16 bp head sequence at the 5′ end (Herbold et al., 2015). The following program was used for amplification: 94°C for 5 min followed by 10 cycles of 94°C for 30 s, 52°C for 45 s, and 72°C for 45 s, and a single step of final elongation at 72°C for 10 min. Following this barcoding PCR step, the samples were purified using ZR-96 DNA Clean-up Kit^TM^ (Zymo, Irvine, CA, United States), quantified using Quant-iT^TM^ PicoGreen^®^ dsDNA Assay Kit (Thermo Fisher Scientific, Waltham, MA, United States) on a Tecan Safire plate reader (Tecan, Männedorf, Switzerland) and pooled in equimolar amounts of 20 × 10^9^ copies per sample. Library preparation and sequencing services were provided by Microsynth (Balgach, Switzerland). The library was prepared by adaptor ligation and PCR using the TruSeq Nano DNA Library Prep Kit (Illumina, Cat FC-121-4001) according to the TruSeq Nano protocol (Illumina, FC-121-4003), but excluding the fragmentation step. Sequencing was performed on a MiSeq platform (Illumina, San Diego, CA, United States). The MiSeq was run in the 2 × 300 cycle configuration using the MiSeq Reagent kit v3 (Illumina, San Diego, CA, United States). The raw sequence data were deposited into the NCBI Short Read Archive under BioProject accession number PRJNA4 32667.

### Construction of Hidden-Markov Models for Filtering Non-*nifH* Reads and for Aligning *nifH* Sequences

Non-*nifH* reads were filtered out using four Hidden-Markov Models (HMMs). One model (hmm_nuc_1160_*nifH*.hmm) was based on 1160 nucleotide sequences from the *nifH*_2014April04.arb database (Heller et al., 2014), with sequence data matching the amplification region of Ueda19F–R6 primer pair (the longest amplicon in our test). Three other models (bchX.hmm, chlL-bchL.hmm, and Zehr_2014_1812genomes_*nifH*_AA.hmm) were based on amino acid sequences. The model Zehr_2014_1812genomes_*nifH*_AA.hmm was based on 1812 aligned *nifH* amino acid sequences from sequenced genomes, obtained from the *nifH*_2014April04.arb database. The model bchX.hmm included 79 amino acid sequences from the NCBI Entrez Protein Cluster chlorophyllide reductase iron protein subunit X (*bchX*, PCLA_924109; also useful in discriminating against the chromosome partitioning protein (*parA*) sequences), and chlL-bchL.hmm included 250 amino acid sequences from two light-independent protochlorophyllide reductase iron-sulfur ATP-binding proteins (*chlL*, CHL00072 and *bchL*, PCLA_858313, PCLA_3385405). Because *bchX* and *chlL*-*bchL* sequences tend to be more similar to *nifH* sequences than to each other, an attempt to generate a single HMM covering all three homologs but excluding *nifH* was unsuccessful. The sequences for the HMMs hmm_nuc_1160_*nifH*.hmm, bchX.hmm, and chlL-bchL.hmm were aligned using MAFFT (V7.271; Katoh and Standley, 2013), employing the L-INS-i algorithm, and then individual HMMs were built from each multiple sequence alignment using the hmmbuild in HMMER V3.1 (Mistry et al., 2013). For convenience, a single HMM database (*nifH*_chlL_bchX.hmm) was generated from the three amino-acid based HMMs using command hmmpress in HMMER.

### NifMAP – An Automated Pipeline for Analyzing *nifH* Amplicon Reads

**FIGURE 1:**
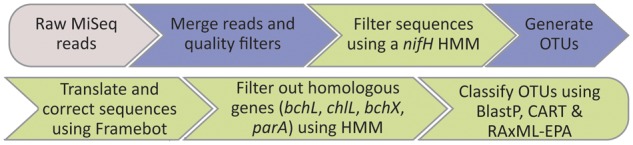
Schematic overview of NifMAP – *NifH* MiSeq Illumina Amplicon Analysis Pipeline. Pipeline begins with raw MiSeq reads from desired sequencing facility (depicted in gray). Steps shaded in blue represent standard processing steps, while steps shaded in green represent steps specific for processing *nifH* sequences, introduced in this work.

To process the raw MiSeq amplicon reads, we devised the following pipeline (**Figure 1**): (1) Paired raw MiSeq reads were assembled into contigs using QIIME’s join_paired_ends.py (Caporaso et al., 2012). (2) The merged contigs were filtered against the nucleotide-based HMM (hmm_nuc_1160_*nifH*.hmm) using hmmsearch command in HMMER. All reads passing through this filtering step were accepted for the next step, irrespective of the model match score. (3) Sequences were chimera-checked and clustered using the UPARSE pipeline (Edgar, 2013). First, contigs were dereplicated with the -derep_fullength command and singleton unique sequences were removed. OTU centroids were then determined with the -cluster_otus command (using 3% radius). Abundances of OTUs were determined by mapping the filtered contigs (prior to dereplication) to OTU centroids using the -usearch_global command (at a 0.97% identity, hereafter referred to as OTU_97_). (4) Translation into amino acids and (potential) frameshift corrections were done using FrameBot (Wang et al., 2013) against the *nifH* protein reference set. (5) Homologous genes to the *nifH* gene (*bchX, chlL, bchL*, and *parA*) were filtered out against the HMM *nifH*_ChlL_bchX.hmm (see above) using the hmmscan command in HMMER. Only OTU representatives that scored highest against the *nifH* model compared with the bchX and chlL-bchL models were retained. (6) OTU classification and phylogenetic placement: the remaining OTU representatives were classified using BLASTP (Camacho et al., 2009) against the RefSeq database (Pruitt et al., 2005). In addition, OTU representatives were aligned using hmmalign to the HMM Zehr_2014_1812genomes_*nifH*_AA.hmm and assigned to phylogenetic clusters of *nifH* using Classification And Regression Trees (CART; Frank et al., 2016). OTU representatives were also placed on a base tree using the Evolutionary Placement Algorithm (EPA) implementation in RAxML (Stamatakis, 2014). The base tree was generated as follows: (1) All entries containing amino acid sequence information for the *nifH* gene in the *nifH*_2014April04.arb database were extracted. These included 1971 entries from genomes and 39,258 entries from non-genome sequencing sources. (2) All sequences shorter than 133 AA were filtered out and the remaining sequences were dereplicated. (3) The remaining sequences were clustered at a clustering threshold of 90% identity using CD-HIT (Fu et al., 2012). In addition, the sequences used for constructing the *bchX* and *chlL*-*bchL* HMMs were clustered at a threshold of 80% identity using CD-HIT and merged with the clustered *nifH* sequences. The combined dataset was then aligned using MAFFT L-INS-i against the aligned collection of 1812 amino acid sequences of genomic origin used for constructing the HMM Zehr_2014_1812genomes_*nifH*_AA.hmm, in order to maintain an alignment compatible with the *nifH*_2014April04.arb database. Lastly, a bootstrapped maximum likelihood tree based on a CAT model was reconstructed using RAxML. For placing the OTU representatives on the base tree, the sequences were aligned using MAFFT against the alignment used for constructing the base tree and then added to the base tree using RAxML, employing the EPA. Steps 1 and 3 of the pipeline are identical to the procedures described in Herbold et al. (2015), while steps 2, 4, 5, and 6 are new for this work. The HMMs, base tree and shell script for reproducing steps 2, 4, 5, and 6 are publicly available at https://github.com/roey-angel/NifMAP.

### Design and Testing of a *nifH* Mock Community

A *nifH* mock community was developed to estimate variations in sequencing quality amongst runs and cross-contamination within a run (Bokulich et al., 2016). This mock community was comprised of DNA from the following six diazotrophic species: *Anabaena torulosa* (Carm.) Lagerh. (Cyanobacteria) Algae Collection Vienna (ASW 01028), *Desulfosporosinus acidophilus* strain SJ4^T^ (DSM22704) (Firmicutes) (Alazard et al., 2010), *Kosakonia sacchari* (Gammaproteobacteria) (in house strain, 16S rRNA gene sequence 99.89% identical (1105 bp) to the 16S rRNA gene sequence of *K. sacchari* SP1^T^ (Chen et al., 2014), *Mesorhizobium loti* strain R7A (Alphaproteobacteria) (Kelly et al., 2014), *Nostoc microscopicum* Vaucher (Cyanobacteria) Algae Collection Vienna (ASW 01020), and *Telmatospirillum siberiense* 26-4b1T (Alphaproteobacteria) (Hausmann et al., 2018). DNA was extracted using Qiagen DNeasy Blood and Tissue Kit (Qiagen) according to the manufacturer’s instructions except that 200 μl of culture was first transferred to Lysing Matrix A tube and cells were disrupted using bead beating (30 s at 4 m s^-1^; FastPrep-24, MP Biomedicals, Santa Ana, CA, United States) after adding PBS and AL buffers. DNA was used for PCR amplification of the *nifH* gene using primers Ueda19F–R6 as described above. PCR products were cloned using TOPO TA Cloning Kit (Thermo Fisher Scientific, Waltham, MA, United States). One clone containing *nifH* gene fragment from each strain was used for colony PCR amplification using primers M13, flanking the insert region (Thermo Fisher Scientific, Waltham, MA, United States). PCR was performed with the following mixture: 5 μl of 10× DreamTaq Green Buffer, 2 mM MgCl_2_, 0.2 mM of each nucleotide dNTP mixture, 0.05 μg μl^-1^ of BSA, 1.25 U of DreamTaq Green DNA Polymerase (all from Thermo Fisher Scientific, Waltham, MA, United States) and 1 μM of each primer and the following conditions for amplification: 94°C for 5 min followed by 30 cycles of 94°C for 60 s, 55°C for 60 s, and 72°C for 60 s, and a single step of final elongation at 72°C for 10 min. Following amplification and purification with ZR-96 DNA Clean-up Kit^TM^ (Zymo, Irvine, CA, United States), PCR products were quantified using Quant-iT^TM^ PicoGreen^®^ dsDNA Assay Kit (Thermo Fisher Scientific, Waltham, MA, United States) on a Tecan Safire plate reader (Tecan, Männedorf, Switzerland) and pooled in equal and also tiered proportions. These mixtures were used as PCR templates for MiSeq sequencing. Each mock community mixture was amplified and sequenced in triplicate using primers Ueda19F–R6, IGK3–DVV, and F2–R6 as described above. The information for generating the mock community can be found at the Mockrobiota repository^1^ under mock-27 and mock-28.

### Richness and Diversity Estimates

Richness was estimated using number of observed OTUs, while diversity was estimated using the inverse Simpson metric:

$$\begin{array}{c} 1/D=1/\sum_{i=1}^{R}P_{i}^{2} \end{array}$$

where *R* represents the number of OTUs in a sample and *P_i_* is the proportional abundance of each OTU. For each sample, both richness and diversity were estimated using a bootstrapped method by iterative subsampling (1000 iterations) to minimum read-depth (after dropping the lowest 15th percentile samples).

### Quantitative PCR Assays

Quantitative PCR (qPCR) reactions were performed on a C1000 Touch thermocycler equipped with a CFX96 Real Time System and the data were processed using CFX Manager software (all from Bio-Rad, Hercules, CA, United States). For all reaction plates, serially diluted standards containing known quantities of DNA copies of the target gene (ranging between 4.2 × 10^1^ and 4.2 × 10^7^) were used for establishing quantitative calibration curves. The standard was generated using a cloned fragment of the *nifH* gene from *Didymococcus colitermitum* TAV2 (ATCC BAA-2264; Wertz et al., 2012). A SYBR^®^ Green I-based assay for *nifH* was established as follows: each reaction was 20 μl in volume and contained 10 μl of SYBR Green JumpStart Taq ReadyMix (Bio-Rad, Hercules, CA, United States), 3 mM MgCl_2_, 0.4 ng μl^-1^ BSA (Thermo Fisher Scientific, Waltham, MA, United States), 1.4 μM of each primer, and 2 μl of template. The program used was: 95°C for 5 min, followed by 45 cycles of 95°C for 30 s, 52°C for 45 s for annealing, 72°C for 30 s for extension, and 78°C for 10 s for signal acquisition. The reliability of quantification was evaluated using a melting curve from 55 to 95°C. A correction for the *nifH* copy numbers was done for each sample separately by excluding the fraction of non-*nifH* sequences (i.e., the proportion of reads that were detected as non-*nifH* by the pipeline) from the initial values obtained from qPCR. Data were evaluated using ANOVA on log-transformed data.

## Results and Discussion

### NifMAP **– *N****ifH* MiSeq Illumina Amplicon Analysis Pipeline

As the majority of amplicon based diversity surveys employ the 16S rRNA gene as a phylogenetic marker, there are standardized analysis pipelines and procedures (e.g., Caporaso et al., 2012; Edgar, 2013; Kozich et al., 2013). This is in stark contrast to amplicon-based surveys targeting functional genes, such as *nifH*. Some of the challenges limiting the development of standardized pipelines for functional genes include co-amplification of non-target genes due to gene homology (Holmes et al., 1995); amino acid-based analyses making sequencing error (generating insertion and deletion errors) more detrimental; and lack of classification methods or databases. Furthermore as every functional gene is different, the methods oftentimes need to be adjusted in a gene-specific manner (Penton et al., 2013). Several methods have been proposed in the literature to tackle issues such as alignment and filtering (Fish et al., 2013), correct conversion to amino acids (Wang et al., 2013) and classification (Dumont et al., 2014; Frank et al., 2016), yet complete pipelines for analyzing environmental functional genes are still lacking. Recently, Gaby et al. (2017) published a perl-based pipeline for OTU clustering and inference of taxonomic affiliation through BLAST of *nifH* amplicon data; however, this pipeline did not employ the use of HMMs to filter out homologous genes.

We developed a bioinformatics pipeline for processing *nifH* reads derived from the MiSeq Illumina sequencing platform called “*NifH* MiSeq Illumina Amplicon Analysis Pipeline” – NifMAP, which uses HMMs to filter out homologous genes and taxonomically classifies sequences using three different approaches. In the first step, the sequences are filtered against nucleic acids based HMMs to remove non-target, non-homologous sequences. Subsequently, sequences are clustered based on 97% identity to the centroid sequence, translated into amino acids, and corrected for possible frame shifts. The OTU representatives are screened against specific HMMs in order to filter out *nifH*-homolog sequences (namely *bchX, chlL, bchL*, or *parA*). The use of HMMs ensures a highly specific and sensitive method to detect and remove non-target sequences that is orders of magnitude faster than filtering based on taxonomic or phylogenetic affiliation. Finally, the remaining sequences are classified taxonomically to their closest relatives and their phylogenetic cluster (**Figure 1**). We chose a combined classification approach using several independent methods, providing information on both the closest taxonomic relative of the queried sequence in addition to its placement in a phylogenetic cluster, because the two classification approaches might occasionally disagree (Frank et al., 2016). Our pipeline was evaluated with a *nifH* mock community and a diverse collection of environmental samples (including roots and rhizosphere samples, BSCs and estuarine water samples).

### Evaluating Coverage of *nifH* Primer Pair Combinations

Across numerous environments, the distribution of the *nifH* gene has been evaluated using various primer pair combinations (e.g., Ueda et al., 1995; Farnelid et al., 2011; Collavino et al., 2014). Although there have been some *in silico* investigations evaluating the performance of these primer pairs along with suggestions as to the best combination(s) (Gaby and Buckley, 2012), these primer pairs have yet to be thoroughly evaluated and tested in the wet-lab, especially when considering samples from highly diverse environments such as soils. To that end, we evaluated the performance of 10 different *nifH* primer combinations. The primer pairs consisted of all possible combinations of three forward primers (Nh21F, Ueda19F, and F2) and three reverse primers (*nifH*1, *nifH*3, and R6) (**Table 1**) as described previously (Gaby and Buckley, 2012), along with the combination IGK3 and DVV. These primers were chosen due to their presumed high coverage (**Table 1**) and ability to generate amplicons in lengths that are suitable for MiSeq sequencing (200–500 bp). Extracted DNAs from beech forest and meadow soil were initially used in a pilot study to evaluate these combinations. The initial criterion was generation of a correctly sized PCR fragment. Only three primer pairs produced PCR fragments of the correct size: F2–R6 (358 bp), Ueda19F–R6 (394 bp), and IGK3–DVV (454 bp) without unspecific bands (data not shown). Primer pairs F2–R6 and IGK3–DVV have been used in the past for analyzing diazotrophic communities (Gaby and Buckley, 2012), while this was the first time that the Ueda19F–R6 combination was used.

### Assessing *nifH* Primer Pair Coverage Using a Mock Community

In order to assess potential primer biases of these three primer combinations, their performance was tested on constructed *nifH* mock communities containing six phylogenetically diverse members. Mock communities, i.e., a defined mixture of known microbial strains, have become a standard tool for benchmarking different aspects of high-throughput sequencing techniques, for example, base calling error, variation between indecent runs and cross contamination (Schloss et al., 2011; Bokulich et al., 2016; Singer et al., 2016). In addition, mock communities can be used to assess amplification bias of different primers. Until now nearly all established mock communities were designed for 16S rRNA gene surveys, while there is a growing need to develop mock communities for other (functional) genes.

Two mock communities were generated using six clones containing *nifH* gene fragments of diazotrophic cultures of even and tiered proportions (**Table 2**). Prior to pooling the clones, each insert was confirmed to perfectly match the used primers based on Sanger sequencing (data not shown). The *nifH* genes in each mock community were amplified and sequenced in triplicate using all three primers pairs, in order to assess the ability of the primers to reconstruct the diazotrophic community structure. Across all three primer pairs, sequencing produced 664–7883 reads per sample after quality filtering, which were classified into 6 (primers Ueda19F–R6, and F2–R6) and 7 (IGK3–DVV) OTUs. OTUs 1–6 were confirmed to match the mock community members (**Table 2**), while OTU 7 was a contaminant identified as a *Geobacter* sp. and comprised of a single read in two mock community replicates amplified using the primer pair IGK3–DVV (data not shown). Sequencing exhibited a high degree of reproducibility among replicates as indicated by the low standard errors (**Table 2**), but showed deviations from the expected read distribution in the even and tiered mock communities. Using primers F2–R6, over 90% of the reads were affiliated to *M. loti* and about 6% were affiliated to *K. sacchari*, while other members of the mock community (including two cyanobacteria) were nearly not detected. This is despite perfect *in silico* primer matching to the *nifH* sequences of the mock community members.

**Table 2:** Relative proportion of the expected and observed reads for an even and tiered *nifH* gene mock community for the different primer pairs.

Mock community composition	Expected distribution of reads		Observed distribution of reads					
			Ueda19F–R6		IGK3–DVV		F2–R6	
Members	Even	Tiered	Even	Tiered	Even	Tiered	Even	Tiered
(1) *D. acidophilus* strain SJ4^T^ (Firmicutes, cluster IC)	16.7%	10%	33 ± 0.9%	23 ± 1.0%	48 ± 1.0%	31 ± 0.5%	0.5 ± 0.0%	0.0 ± 0.0%
(2) *M. loti* strain R7A (Alphaproteobacteria, cluster IJ)	16.7%	20%	16 ± 0.4%	21 ± 0.2%	18 ± 0.4%	25 ± 0.7%	90.7 ± 1.1%	93.1 ± 0.7%
(3) *N. microscopicum* Vaucher ASW 0120 (Cyanobacteria, cluster IB)	16.7%	40%	12 ± 0.4%	34 ± 0.4%	14 ± 0.4%	36 ± 0.8%	0.7 ± 0.2%	0.3 ± 0.1%
(4) *A. torulosa* (Carm.) Lagerh. ASW 01028 (Cyanobacteria, cluster IB)	16.7%	5%	16 ± 0.9%	4 ± 0.4%	19 ± 1.4%	5 ± 0.8%	0.0 ± 0.0%	0.0 ± 0.0%
(5) *K. sacchari* (Gammaproteobacteria, cluster IG)	16.7%	20%	12 ± 0.2%	14 ± 0.5%	1 ± 0.1%	2 ± 0.3%	5.7 ± 0.3%	5.7 ± 0.5%
(6) *T. siberiense* strain 26-4b1T (Alphaproteobacteria, cluster IJ)	16.7%	5%	11 ± 0.4%	4 ± 0.5%	1 ± 0.0%	0 ± 0.0%	0.9 ± 0.1%	0.2 ± 0.0%

In contrast, all mock community members could be recovered using primers Ueda19F–R6 and IGK3–DVV. The bias from the expected distribution (even and tiered) was greater in the IGK3–DVV primers compared to the Ueda19F–R6 primers (**Table 2**). Specifically, *D. acidophilus* was consistently overrepresented in both the even and tiered mock communities for both primer pairs, while *K. sacchari* and *T. siberiense* were underrepresented. *D. acidophilus, K. sacchari*, and *T. siberiense* were particularly biased against using the primer pair IGK3–DVV. Biases in the composition of microbial communities resulting from differential amplification of templates by PCR especially when using degenerate primer pairs, are well documented through high-throughput sequencing of 16S rRNA gene as well as functional gene mock communities (e.g., Caporaso et al., 2011; Schloss et al., 2011; Pinto and Raskin, 2012; Pelikan et al., 2016). As in 16S rRNA gene amplicon sequencing (Schloss et al., 2011), our results therefore stress the need for including mock community samples that are specific to the target gene of interest in sequencing experiments for assessing the extent of primer biases. Furthermore, when mock communities are sequenced along with environmental samples on one sequencing run, it allows verifying the reproducibility of different runs.

### Assessing *nifH* Primer Pair Performance Using Environmental Samples

The specificities of the primer pairs Ueda19F–R6, IGK3–DVV, and F2–R6 were further evaluated by amplicon sequencing of environmental samples. In addition to the above-mentioned temperate soil samples, we extended our investigation to a diverse collection of terrestrial samples (such as BSCs, rhizosphere and root samples) along with two estuarine water samples. A total of 424,234 raw Illumina paired-end reads were generated across these primer pairs: Ueda19F–R6 (156,476 reads), IGK3–DVV (202,300 reads), and F2–R6 (65,458 reads). The raw reads were processed through our automated pipeline NifMAP (see section “Materials and Methods” and **Figure 1**). After contig assembly, the merged reads were filtered using the nucleic acids based HMM, which filtered out different proportions of reads depending on the used primer combinations. Most reads were removed in the F2–R6 primer pair (ca. 7.5%) followed by Ueda19F–R6 (ca. 2.0%) and IGK3–DVV (ca. 0.3%). Investigating these non-*nifH* sequences using BLASTN against the Nr database gave no significant matches. The remaining reads were clustered into OTUs with the IGK3–DVV primer pair generating the most reads (89,453) that were clustered into 884 OTU_97_, followed by Ueda19F–R6 (76,897 reads; 528 OTU_97_) and F2–R6 (45808 reads; 763 OTU_97_). OTU representatives were converted to amino acid sequences, but in no case did we observe that frame shift correction was needed. This is in line with previous reports showing a particularly low indel error rate for Illumina MiSeq technology (Schirmer et al., 2015). Next, filtering of the OTU sequence representative via HMMs of homologous genes to *nifH* showed that all primer pairs and samples produced reads, and consequently OTUs, which were not classified as *nifH*, but rather as these homologous genes. Most of these homologs were classified as *chlL-bchL*, followed by *bchX*, and only a minority of sequences were classified as *parA*.

**FIGURE 2:**
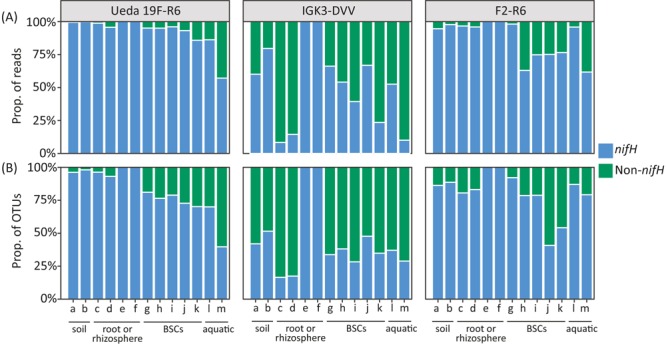
Performance of the *nifH* primer pairs in amplifying *nifH* and non-*nifH* (homologous) genes across environmental samples based on proportion of reads **(A)** and OTUs **(B)**. The environmental samples include: (a) beech forest soil; (b) meadow soil; (c) rhizosphere and (d) root samples of *Arrhenatherum elatius*; (e) rhizosphere and (f) root samples of *Oryza sativa*; (g) coastal, sub-arctic biological soil crust (BSC); (h) temperate BSC; (i) high alpine BSC; (j) semiarid BSC; (k) arid BSC; estuarine samples from the (l) Great Belt and (m) Roskilde Fjord. More details on the samples can be found in the Section “Materials and Methods.” Reads or OTUs classified as homologous genes (such as *bchX, chlL, bchL*, and *parA*) are summarized as “non-*nifH*.”

While the amplification of homologous genes was observed in all three primer pairs, striking differences amongst the primer pairs were seen in the proportion of reads and OTUs classified as homologs rather than as *nifH* (**Figure 2**). Across many environmental samples, the IGK3–DVV combination exhibited the highest proportion of non-specific products with on average 48% non-*nifH* OTUs and up to 92% non-*nifH* reads (**Figure 2**, green bars). The primer pairs F2–R6 and the new tested combination, Ueda19F–R6, amplified less non-*nifH* sequences across many of the investigated samples, with an average of 7.5 and 13% for primers Ueda19F–R6 and F2–R6, respectively. Our analysis shows not only large differences in the extent of non-specific products amongst the different primer pairs (*P* < 0.01), but also amongst environmental samples when the same primer pair was used (*P* = 0.02). The most striking difference was detectable amongst the samples of rice and tall oat-grass. Using primers IGK3–DVV, nearly all reads and OTUs in the rice samples were classified as *nifH*-related (**Figure 2**, samples “e, f”), while in the tall oat-grass samples only around 10% of them were identified as *nifH*-related (**Figure 2**, samples “c, d”). Similar trends can be seen for the crust and water samples, though to a lesser extent. The problem of co-amplification of non-target homologous or non-homologous genes during PCR is well documented in the literature, particularly when amplifying functional genes (Holmes et al., 1995). Unfortunately, this phenomenon can erroneously inflate diversity estimates and population size when left unnoticed. Our analysis clearly shows that different primer pairs can have striking differences in amplification specificities and that this should be taken into consideration when designing a genetic survey.

### Comparing Alpha-Diversity Metrics Across the Primer Pairs

We calculated the number of observed OTUs (richness estimate) and the inverse Simpson metric (diversity estimate) from each sample type and with the three different primer pairs. On average, similar numbers of *nifH* OTUs per sample were detected using each of the three primer pairs Ueda19F–R6 (49 ± 3); IGK3–DVV (39 ± 3); and F2–R6 (49 ± 4), while the inverse Simpson index was somewhat lower using primers Ueda19F–R6 (9.5 ± 0.5) compared to the other two primer pairs, IGK3–DVV (14.0 ± 1.5) and F2–R6 (14.0 ± 1.8) (Supplementary Figure S1) indicating no clear advantage of one primer pair in capturing higher richness. A general agreement amongst the primer pairs was seen across the different sample types regarding richness and diversity patters. For example, the beech forest and meadow soils harbored richer and more diverse communities than the crust and water samples. Nevertheless, there were some differences amongst the primer pairs with regards to richness and diversity estimates. Most notably primer pair F2–R6 yielded higher richness and diversity estimates for rice rhizosphere and root samples (Supplementary Figure S1, samples “e, f”), but lower for many of the crust samples (Supplementary Figure S1, samples “g–k”), compared to primer pairs IGK3–DVV and Ueda19F–R6.

### Taxonomic Description of the Diazotrophic Community Using Multiple Classification Approaches Across the Primer Pairs

The classification of *nifH* genes is not an easy task due to a lack of taxonomic affiliation to functional gene sequences (Gaby and Buckley, 2011), poor reflection of the *nifH* phylogeny to the 16S rRNA gene phylogeny (Zehr et al., 2003), and insufficient information in *k*-mer frequencies to differentiate amongst the subclusters (Frank et al., 2016) as was also observed with *pmoA* sequences (Dumont et al., 2014). As such, we propose that a combined classification approach, using several independent methods might be more beneficial for characterizing *nifH* sequence datasets. Several approaches have been described in the literature for rapid, accurate and biologically meaningful classification of functional gene sequences in general and *nifH* in particular, including nearest neighbor identification in pairwise alignment using FrameBot (Wang et al., 2013), lowest common ancestor parsing of BLAST bit scores using MEGAN (Huson et al., 2007; Dumont et al., 2014), or CART (Frank et al., 2016). Here, we used a combined approach in our pipeline and describe the taxonomic affiliation of the sequences using closest relative via BLASTP, *nifH* cluster and subcluster affiliation using CART (Frank et al., 2016), and the EPA (Berger et al., 2011) for phylogenetic tree generation.

**FIGURE 3:**
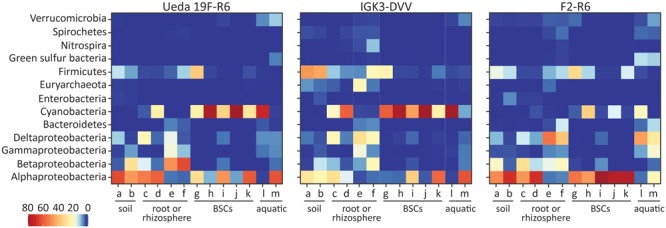
Taxonomic classification of *nifH* sequences based on a BLASTP search for the different primer pairs and environmental samples. Colors of the heatmap indicate the mean relative abundance of the taxonomic classes from each sample. The environmental samples include: (a) beech forest soil; (b) meadow soil; (c) rhizosphere and (d) root samples of *Arrhenatherum elatius*; (e) rhizosphere and (f) root samples of *Oryza sativa*; (g) coastal, sub-arctic biological soil crust (BSC); (h) temperate BSC; (i) high alpine BSC; (j) semiarid BSC; (k) arid BSC; estuarine samples from the (l) Great Belt and (m) Roskilde Fjord.

**FIGURE 4:**
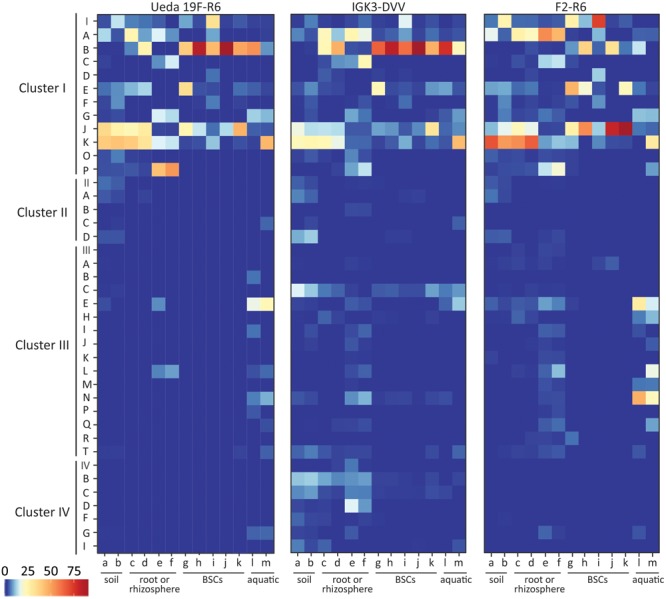
Taxonomic classification of *nifH* sequences based on CART for the different primer pairs and environmental samples. Colors of the heatmap indicate the mean relative abundance of the subclusters in each sample. The environmental samples include: (a) beech forest soil; (b) meadow soil; (c) rhizosphere and (d) root samples of *Arrhenatherum elatius*; (e) rhizosphere and (f) root samples of *Oryza sativa*; (g) coastal, sub-arctic biological soil crust (BSC); (h) temperate BSC; (i) high alpine BSC; (j) semiarid BSC; (k) arid BSC; estuarine samples from the (l) Great Belt and (m) Roskilde Fjord.

**FIGURE 5:**
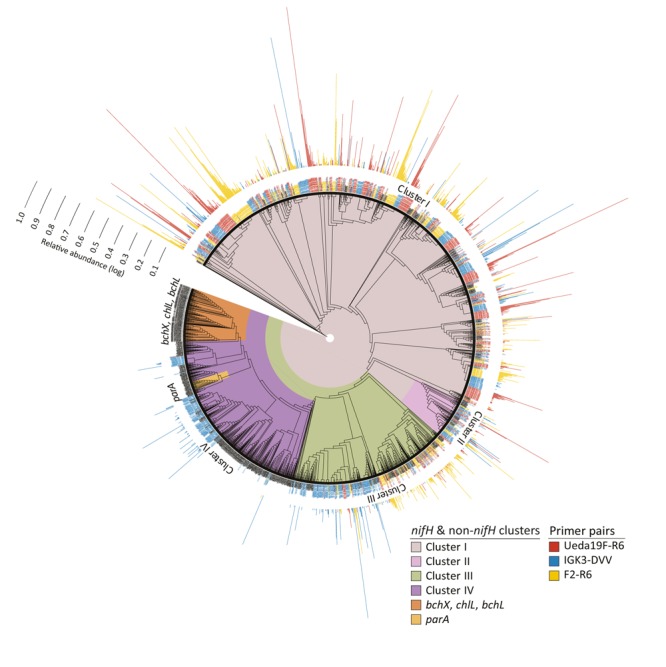
Unrooted RAxML-EPA tree of *nifH* deduced amino acid sequences (with at least 133 positions). The *nifH* and non-*nifH* (homologous genes) clusters are represented in different colors. Representative sequences from the “*nifH*_2014April04.arb” database (Heller et al., 2014) are depicted in black. The phylogenetic placements of OTU representatives from this study for the three primer pairs are depicted at the terminal nodes: sequences obtained with primer pair Ueda19F–R6 are shown in red, IGK3–DVV-derived sequences in blue, and F2–R6-derived sequences in yellow. Outermost bars represent the relative abundance [log(*x*% + 1)] of each OTU.

A representative from each OTU was classified using the best matching hit in BLASTP (**Figure 3**), CART (**Figure 4**), and the EPA for phylogeny (**Figure 5**). The BLASTP classifies sequences based on their closest relative. This classification yielded an overall agreement amongst the three primer pairs, with mainly members of the Alphaproteobacteria, Betaproteobacteria, Deltaproteobacteria, Cyanobacteria, and Firmicutes populating the diazotrophs across the environments (**Figure 3**). However, the relative abundance of these taxonomic groups varied amongst the investigated primer pairs. For example, Cyanobacteria were underrepresented by primer pair F2–R6 in comparison to the other two primer pairs (in concordance with the performance of primers F2–R6 amplifying the mock community). Furthermore, the relative abundance of Alphaproteobacteria derived in PCRs with primer pair IGK3–DVV was lower in some samples than in PCRs with the other two primer combinations, and Firmicutes were comparatively underrepresented by primer pair Ueda19F–R6 in some samples (**Figure 3**). Some taxa (such as the Green Sulfur Bacteria) were completely missing with the IGK3–DVV primer pair, while Euryarchaeota were missing in the Ueda19F–R6 primer pair and at low relative abundance with primer pair F2–R6 (**Figure 3**).

The CART classification assigns sequences to four clusters of *nifH*, termed clusters I–IV with subclusters (Zehr et al., 2003; Raymond et al., 2004). The majority of the sequences amongst all primer pairs were classified into cluster I based on the CART classification (**Figure 4**). This cluster is known to reflect 16S rRNA-based phylogeny relatively well (Zehr et al., 2003) and in many environments is the largest and most ecologically important diazotroph cluster (Gaby and Buckley, 2011). Within cluster I, differences in the relative abundances of certain defined clusters can be observed, some of which are in agreement with the BLASTP results. For instance, the relative abundance of sequences in cluster IB (affiliated with Cyanobacteria) derived from PCRs with primer pair F2–R6 was lower than from the other two primer combinations. Furthermore, less sequences assigned to cluster IJ and IK (containing Alphaproteobacteria) were derived from primer pair IGK3–DVV than in the other two primer pairs (**Figure 4**). The same trend was observed when the sequences were analyzed via BLASTP.

As expected, only a minority of the sequences were affiliated with clusters II, III, and IV. All three primer pairs amplified sequences from the same subclusters in clusters II–IV, with some exceptions in clusters III and IV, indicating preferential amplifications of subclusters amongst these primer sets (**Figure 4**). Most notably the IGK3–DVV primer pair amplified many sequences from clusters IIIC (affiliated with Clostridia) as well as from clusters IVB and IVC. In contrast, primer pairs Ueda19F–R6 and F2–R6 amplified very few sequences from clusters IIIC, IVB and IVC. A close examination of the sequence region matching the R6 primer showed that the primer has indeed two mismatches at the 3′ end of several Clostridia sequences affiliated with cluster IIIC (with residues CT instead of GC).

The phylogenetic placement of OTU representatives suggests that the sequences are in clusters I–IV (**Figure 5**). It showed a nearly identical coverage of clusters I and II by all three primer pairs, thus indicating that all three primer pairs cover equally well the diazotrophic diversity in these clusters. Although all three primer pairs captured sequences from cluster III, discrepancies within subclusters were noted, especially with the primer pair IGK3–DVV as compared to F2–R6 and Ueda19F–R6. While primers IGK3–DVV captured many sequences in subcluster IIIC, primers F2–R6 and Ueda19F–R6 captured many from subclusters IIIE, IIIL, IIIM, and IIIN, which were largely missed out by primers IGK3–DVV. Cluster IV sequences were nearly only captured by primer pair IGK3–DVV. However, the ecological relevance of cluster IV members is still debatable, as until recently it was considered to be populated only by genes encoding for functions unrelated to N_2_ fixation (Raymond et al., 2004; Zheng et al., 2016). Moreover this cluster includes several *nifH* homologs, which might be more abundant than *nifH* in certain environments. Attempts to use primers that capture sequences from this cluster could inflate datasets with non-target genes.

### Suitability of *nifH* Primers for qPCR Assays

**FIGURE 6:**
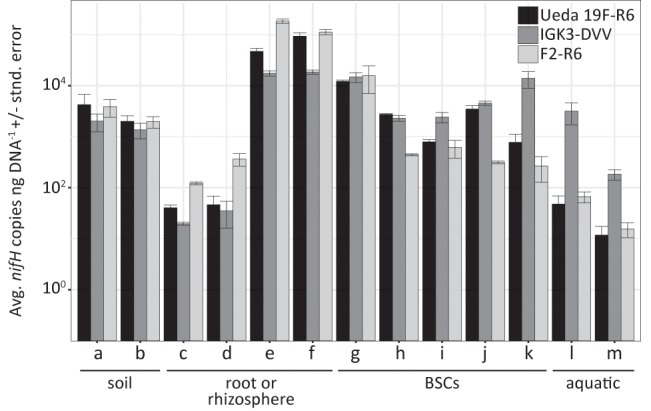
Average *nifH* copies per ng DNA ± standard error across environmental samples based on quantitative PCR using the different primer pairs. The environmental samples include: (a) beech forest soil; (b) meadow soil; (c) rhizosphere and (d) root samples of *Arrhenatherum elatius*; (e) rhizosphere and (f) root samples of *Oryza sativa*; (g) coastal, sub-arctic biological soil crust (BSC); (h) temperate BSC; (i) high alpine BSC; (j) semiarid BSC; (k) arid BSC; estuarine samples from the (l) Great Belt and (m) Roskilde Fjord. Numbers of copies were corrected to exclude non-*nifH* genes that were co-amplified using information obtained from amplicon sequencing of the samples with the specific primer pairs.

The primer pairs F2–R6, IGK3–DVV, and Ueda19F–R6 were also evaluated for their suitability in qPCR assays. Since using degenerate primers incurs a potential template-depended quantification bias, we increased the primer concentration in these reactions to 1.4 μM. This has been recently shown to minimize the effect of this specific bias (Gaby and Buckley, 2017) and has increased efficiency in our assays (data not shown). High assay efficiencies across all three primer pairs – Ueda19F–R6 (97.4%), IGK3–DVV (92.5%), and F2–R6 (90.5%) – were attained (Supplementary Figure S2), illustrating that these primer sets worked almost equally well and therefore can be used for reliable quantification of *nifH* genes and transcripts. In a proof-of-principle experiment, we quantified *nifH* genes in the environmental samples used in this study across all three primer pairs. A caveat when using such quantitative assays is that qPCR works under the underlying assumption that all quantified templates belong to the target gene. As we have demonstrated above, this is not the case for many types of samples when using general *nifH*-targeting primers. Therefore, we corrected for the proportion of *nifH* sequences from the total reads based on the sequencing results (**Figure 6**). Amongst these diverse sample types, the numbers of *nifH* copies per ng DNA ranged between 1.2 × 10^1^ and 1.8 × 10^5^ with a geometric average of 1.3 × 10^3^. Interestingly, no single primer pair gave consistently higher or lower estimations compared to other primer pairs (*P* = 0.5, ANOVA test), further reiterating that all three primer sets worked equally well. Yet, significant differences appeared to be sample-type dependent (*P* < 0.01, ANOVA test). The estimated *nifH* copies per ng DNA were congruent within a given sample amongst the three primer pairs, considering the precision limitations of qPCR (two- to threefold difference between samples; Hospodsky et al., 2010). However, some exceptions to that observation were notable. For instance, primer pairs F2–R6 and Ueda19F–R6 estimated similar copy numbers in estuarine water samples (**Figure 6**, samples “l, m”) and the arid crust sample (**Figure 6**, sample “k”), while primer pair IGK3–DVV estimated an order of magnitude more copies. Furthermore, primers Ueda19F–R6 and IGK3–DVV measured similar copy numbers in the temperate and semiarid BSCs (**Figure 6**, samples “h, j”) while primers F2–R6 measured an order of magnitude less copies. This latter observation could be explained by the fact that primer pair F2–R6 has the tendency to discriminate against cyanobacteria, which would result in a decreased copy number in these cyanobacteria-dominated samples.

## Conclusion

We tested the *nifH*-targeting primer combinations Ueda19F–R6, IGK3–DVV, and F2–R6 for their performance in genetic surveys of diazotrophs, thereby elaborating on the *in silico* analysis of Gaby and Buckley (2012). All primer pairs had a propensity to co-amplify homologs of the *nifH* gene at varying proportions, which is common in many studies of functional gene diversity. Most severe, primer combination IGK3–DVV had the largest tendency to co-amplify these sequences (which are most closely related to cluster IV), with some samples yielding up to ca. 90% homolog sequences. Using a pipeline such as our newly established NifMAP, that permits the detection of non-specific co-amplified reads via specifically designed HMMs, proved to be a useful strategy in filtering out such reads.

When analyzing the specificity of these primers through the use of a mock community along with a diverse collection of environmental samples, we observed some degree of bias amongst the primer pairs. As such, care should be taken when choosing primer pairs for diazotrophic diversity investigations. More specifically, primer pair F2–R6 has a propensity to discriminate against cyanobacteria (amongst others) in our mock communities and environmental samples, yet captured many sequences from subclusters IIIE, IIIL, IIIM, and IIIN. These aforementioned subclusters were largely missing by the primer pair IGK3–DVV, which also tended to discriminate against the Alphaproteobacteria, but amplified many sequences within clusters IIIC (affiliated with Clostridia) and clusters IVB and IVC. Primer pair Ueda19F–R6 exhibited the least bias based on our mock community analysis and successfully captured diazotrophs in cluster I as well as in subclusters IIIE, IIIL, IIIM, and IIIN, but discriminated against Firmicutes (based on BLASTP analysis) and subcluster IIIC. Thus, depending on the investigated environmental sample and the aforementioned primer performance, one should choose the appropriate primer combination. Apart from providing useful analysis protocols and standards for the study of environmental diazotrophs, our work highlights some of the important pitfalls and caveats of studying *nifH*, and potentially other functional genes in the environment. Furthermore, these primer pairs can be used in targeted functional investigations in tandem with activity measurements of N_2_ fixation (Angel et al., 2018) to better understand the ecophysiology of diazotrophs in the environment and could potentially be used to identify target groups for downstream single-cell analyses (Eichorst et al., 2015; Woebken et al., 2015). We hope this knowledge, along with our newly develop pipeline (NifMAP) will aide investigators to better detect and capture diazotrophs in their respective environment.

## Author Contributions

RA, SE, and DW designed the study and participated in writing. RA, CP, and HS extracted DNA and performed the PCRs. MN and HS constructed the mock community. RA, MN, and CH established and tested the analysis pipeline, NifMAP. RA analyzed the data.

## Conflict of Interest Statement

The authors declare that the research was conducted in the absence of any commercial or financial relationships that could be construed as a potential conflict of interest.

## Footnotes

1 http://github.com/caporaso-lab/mockrobiota/
