# Supplementary material for: Evaluation of Primers Targeting the Diazotroph Functional Gene and Development of NifMAP – A Bioinformatics Pipeline for Analyzing nifH Amplicon Data
Source: Front Microbiol. 2018 Apr 30;9:703. doi: 10.3389/fmicb.2018.00703 (PMC5936773; doi:10.3389/fmicb.2018.00703)
Supplement: Supplementary file 1 [file Presentation_1.PDF]

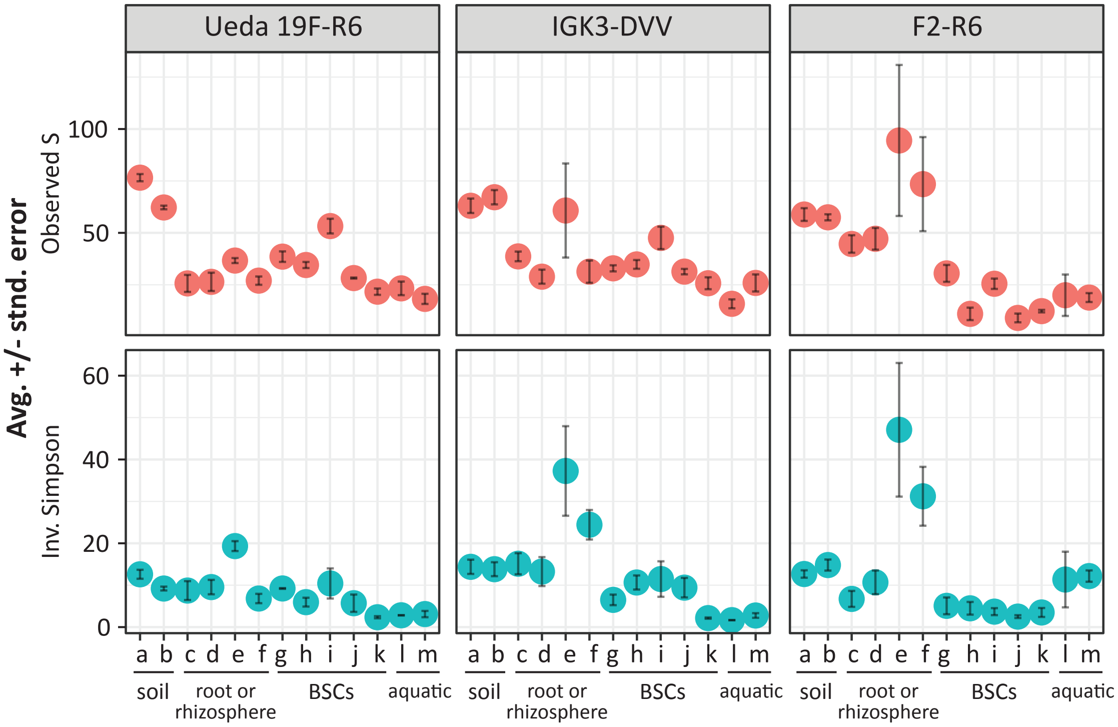

Figure S1. Average +/- standard error for richness (observed S) and diversity (Inv. Simpson) estimates across environmental samples using the primer pairs. Estimates for each sample were calculated using bootstrapped sub-sampling to the 15th percentile lowest read-depth using 1000 iterations.The environmental samples include: (a) beech forest soil; (b) meadow soil; (c) rhizosphere and (d) root surface of *Arrhenatherum elatius*; (e) rhizosphere and (f) root surface of *Oryza sativa*; (g) coastal biological soil crust (BSC); (h) temperate BSC; (i) high alpine BSC; (j) semiarid BSC; (k) arid BSC; estuarines from the (l) Great Beltand and (m) Roskilde Fjord. More details on the sites can be found in the Materials and Methods.

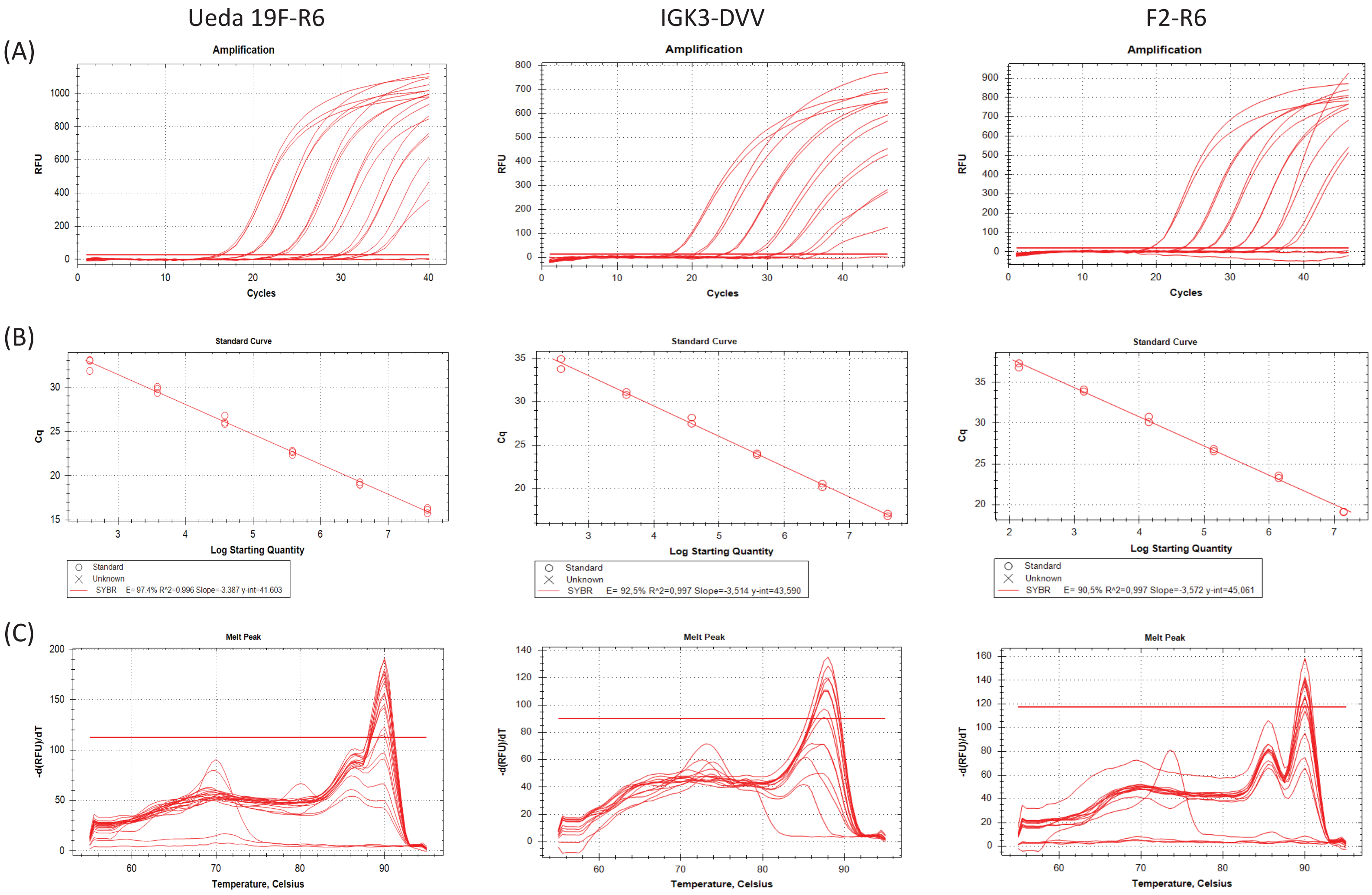

Figure S2. Establishment of *nifH* qPCR assays. qPCR amplification (panels A), standard (panels B) and melting (panels C) curves are depicted for a serially diluted DNA standard for Ueda19F-R6, IGK3-DVV and F2-R6 primer pairs.
